# Factors influencing learners learning interest when using AI chatbots-assisted math leaning in higher education

**DOI:** 10.3389/fpsyg.2025.1716913

**Published:** 2025-12-05

**Authors:** Kang Li

**Affiliations:** School of Economics and Management, Zhejiang University of Water Resources and Electric Power, Hangzhou, China

**Keywords:** AI-supported learning, learning interest, mathematics education, mathematics anxiety, PLS-SEM, self-efficacy, UTAUT

## Abstract

**Introduction:**

This study investigates the determinants of undergraduate learners’ interest in AI-supported mathematics education. Drawing upon the Unified Theory of Acceptance and Use of Technology (UTAUT) and Social Cognitive Theory, a structural model was proposed and empirically tested, incorporating mathematics anxiety, self-efficacy, performance expectancy, and effort expectancy.

**Methods:**

Data were collected from 247 Chinese undergraduates and analyzed using Partial Least Squares Structural Equation Modeling (PLS-SEM).

**Results:**

The findings indicate that performance expectancy, effort expectancy, and self-efficacy are significant predictors of learning interest, with effort expectancy exerting the strongest influence. Self-efficacy also indirectly affects learning interest through its influence on perceptions of the system’s usefulness and ease of use. Contrary to prior research, mathematics anxiety did not significantly predict either self-efficacy or learning interest, suggesting that AI-facilitated environments may buffer negative emotional effects. Academic major moderated the relationship between mathematics anxiety and learning interest, reflecting disciplinary differences in motivational dynamics.

**Discussion:**

This research contributes to theory by integrating motivational constructs into technology acceptance models and extending AI applications to cognitively demanding domains. Practical implications include prioritizing user-centered design and targeted self-efficacy interventions to enhance learner engagement.

## 1 Introduction

The unprecedented acceleration of artificial intelligence (AI) development has transformed numerous sectors of society, marked by milestones such as IBM’s Deep Blue defeating Garry Kasparov and Google’s AlphaGo surpassing human champions in the realm of Go. These advances herald a new era wherein AI technologies ranging from autonomous vehicles to service robots, are increasingly integrated into everyday life (Huang and Rust, 2021; Singh, 2025). Within the educational domain, AI-driven chatbots have emerged as promising tools with the potential to transform teaching, learning, and assessment practices (Chang et al., 2023; Vetrivel et al., 2025). Tools like ChatGPT exemplify this pedagogical shift, offering novel ways to personalize and enhance learning experiences. Consequently, educators in tertiary institutions are beginning to incorporate AI chatbots into classroom instruction and assignment design, anticipating improvements in student engagement and academic outcomes (Chen et al., 2023; Chang et al., 2023; Schönberger, 2024). Despite growing interest, empirical research examining the pedagogical implications of AI chatbots remains limited, particularly concerning their impact on learners’ cognitive and emotional engagement in specific subject domains. Existing studies have predominantly focused on educators’ attitudes or technological adoption models, with insufficient attention paid to students’ psychological responses or motivational dynamics during chatbot-mediated learning especially in subjects such as mathematics, which are often perceived as cognitively demanding and emotionally taxing.

Among the psychological constructs central to learning, self-efficacy and learning interest are consistently found to influence students’ academic behaviors and outcomes (Hanham et al., 2021; Lee et al., 2014; Pan, 2020). Self-efficacy which is defined as the individuals’ belief in their capabilities to execute actions necessary to achieve specific outcomes (Bandura, 1986), has been verified to be positively associated with persistence, cognitive engagement, and achievement in the context of digital learning (Lund and Wang, 2023; Hodges, 2010). Moreover, students’ learning interest which is a key motivational driver that not only affects students’ performance but also influence their long-term educational and career trajectories, has been limited investigated what factors contribute to the formation of students’ learning interest in the perspectives of AI-driven chatbots for math learning (Agyemang et al., 2023; Cho, 2022). More importantly, in the realm of technology acceptance, performance expectancy (PE) and effort expectancy (EE) have been examined to be two major predictors of individual’s intention to adopt new technology. For instance, the unified theory of acceptance and use of technology (UTAUT) has postulated these two determinants impacting on users’ behavioral intention towards the usage of information system (Williams et al., 2015). Subsequently, the importance of these two predictors has been examined to influence on users’ willingness to use AI which were contained in the framework of AI device use acceptance (AIDUA) (Bai and Yang, 2025; Ma and Huo, 2023). Though the significance of these constructs has been well-addressed in prior studies, while the casual relationship among users’ self-efficacy, expectancy and their learning interest was rarely investigated.

Specific in the context of math learning and education, due to the prevalence of math anxiety, students’ self-efficacy and learning interest have been the major concerns (Ashcraft, 2002; Beilock et al., 2010). And studies in the context of higher education related to the math education, the influence of students’ math anxiety on their self-efficacy and their learning interest was seldomly examined with the AI-driven chatbots (Akin and Kurbanoglu, 2011; McMullan et al., 2012). More importantly, cross-cultural studies (e.g., Lee, 2009) suggest that Chinese learners, despite high academic performance, often report low math self-concept and high levels of anxiety. The implications of this emotional barrier when coupled with the affordances of emerging technologies like AI chatbots remain underexplored. This gap has been witnessed by various countries or areas around the whole world. Moreover, based on the studies within the realm of Chinese higher education, this also has been noticed where both technological innovation and academic pressure are intensifying (Wu and Liu, 2021; Xie et al., 2023).

Moreover, the moderating effect of students’ gender on mechanism of their learning interest has been examined in the math learning (Huang et al., 2019; Zamora-Araya et al., 2022). Besides, the influence of students’ major such as natural science or social science on students’ learning interest and their adoption to the AI chatbots has been investigated previously (Hyun and Kim, 2018). However, the potential moderating role of students’ major on the formation of their learning interest has been limited determined.

Against this backdrop, the present study seeks to examine the interplay between math anxiety, self-efficacy, EE, PE, and learning interest in AI chatbot-assisted mathematics learning among Chinese university students. Specifically, we aim to (1) develop a theoretical model grounded in the Unified Theory of Acceptance and Use of Technology (UTAUT) and Social Cognitive Theory, and (2) empirically test the model using Partial Least Squares Structural Equation Modeling (PLS-SEM) with data collected from undergraduate learners.

This paper is structured as follows: The next section reviews the relevant literature and theoretical framework supporting the study. The third section outlines the research methodology, including sample, measurement instruments, and analytical techniques. The fourth section presents the empirical findings, followed by a discussion of the implications for theory and practice. Finally, the study would be concluded by highlighting the study’s contributions, limitations, and directions for future research.

## 2 Literature review

### 2.1 Performance expectancy and effort expectancy in UTAUT

“Performance expectancy” (PE) and “effort expectancy” (EE) are two key determinants of individual technology acceptance, particularly in educational settings. As defined by Venkatesh et al. (2003), PE refers to the degree to which individuals believe that using a technology will enhance their task performance, while EE reflects the perceived ease of use. These constructs draw upon concepts such as “perceived usefulness” and “perceived ease of use” from the Technology Acceptance Model (TAM), but extend them into a broader framework of behavioral intention.

In the context of AI chatbot-supported learning, PE and EE are especially relevant. Students using unfamiliar digital tools must assess not only how helpful these tools are in supporting learning tasks (PE), but also how easy they are to operate (EE). When learners perceive chatbots as both effective and user-friendly, they are more likely to engage with the tool voluntarily, with greater persistence and emotional involvement.

Several studies have supported the role of PE and EE in influencing learner engagement and motivation. For example, Zhang et al. (2023) found that students who perceived AI learning systems as useful and accessible reported stronger academic interest and satisfaction. Similarly, Zhou (2023) reported that usability perceptions significantly influenced students’ emotional responses and behavioral intentions toward AI-enhanced platforms. These findings reinforce earlier research indicating that a favorable cost–benefit evaluation of technology increases its utilitarian value and promotes a positive attitude toward use (Alwahaishi and Snás̃el, 2013; y Monsuwé et al., 2004).

Furthermore, EE is often associated with intrinsic motivational factors, such as autonomy and perceived competence, while PE reflects more extrinsic, outcome-driven expectations (Karahanna and Angst, 2006). In mathematics learning, where cognitive demands are high and emotional barriers such as anxiety are common, these two constructs may shape how students engage with digital tools both emotionally and behaviorally.

Based on these theoretical and empirical insights, PE and EE are incorporated into this study to examine how students’ perceptions of AI chatbot usefulness and usability influence their interest in learning mathematics through such systems.

### 2.2 Math anxiety

Math anxiety is widely acknowledged as one of the most pervasive emotional barriers affecting students’ performance and motivation in mathematics learning. It refers to a feeling of tension, fear, or helplessness that interferes with the manipulation of numbers and problem-solving tasks (Ashcraft, 2002; Wang et al., 2014). According to Wigfield and Meece (1988), math anxiety often stems from prior negative learning experiences and low perceptions of competence, which can lead to long-term disengagement and reduced achievement.

Numerous empirical studies have confirmed that math anxiety negatively influences students’ cognitive performance and motivational states. For instance, Carey et al. (2017) conducted a meta-analysis demonstrating a robust negative correlation between math anxiety and mathematics achievement. This is particularly concerning in higher education, where independent learning is more prominent and emotional self-regulation becomes increasingly essential. Beilock and Maloney (2015) further emphasized that anxiety can impair working memory function during high-stakes problem-solving, thereby reducing not only task performance but also confidence and interest in mathematical tasks.

In the context of digital learning environments, these challenges may be amplified. Without the immediate support of instructors or peers, learners who experience math anxiety may struggle to persist through difficult problems, avoid interacting with intelligent systems, or disengage entirely (Wang et al., 2014). This is especially in AI-supported mathematics learning, where the perceived novelty and complexity of the system may heighten psychological distance and apprehension, limiting students’ willingness to explore (Namaziandost and Rezai, 2024).

Furthermore, research has increasingly highlighted the links between math anxiety and other key psychological factors such as self-efficacy and learning interest. According to Wigfield and Eccles (2000), negative emotions such as anxiety reduce students’ perceptions of control and competence, which in turn diminishes their intrinsic motivation. Students with higher math anxiety tend to report lower levels of task-specific confidence, reduced curiosity, and limited interest in mathematics-related activities (Carey et al., 2017; Ashcraft, 2002). These effects are particularly pronounced among university students with prior math difficulties or limited exposure to technology-enhanced learning environments. In light of these findings, math anxiety is considered a central variable in this study. Its influence on both self-efficacy and learning interest may help explain individual differences in students’ emotional engagement with AI chatbot-supported mathematics learning.

### 2.3 Self-efficacy and learning interest

Self-efficacy, a key component in Bandura’s social cognitive theory, refers to individuals’ judgments about their ability to perform specific tasks or activities (Bandura, 1997). It is widely recognized as a central factor in shaping students’ motivation, persistence, and emotional engagement (Zimmerman, 2000). In educational settings, particularly in mathematics, self-efficacy influences how students interpret challenges, cope with failure, and sustain interest throughout learning tasks (Du and Liu, 2017).

In mathematics learning, self-efficacy has been linked to the selection of strategies, willingness to attempt complex problems, and sustained attention to tasks. Students with high math-specific self-efficacy tend to display greater intrinsic interest and stronger goal orientation, while those with low efficacy often report avoidance behaviors and emotional disengagement (He and Qi, 2018; Wang et al., 2014). These patterns are particularly evident in higher education, where autonomous learning is the norm and self-directed motivation becomes essential.

When technology is introduced, the role of self-efficacy becomes even more prominent. Learners must not only manage cognitive demands but also navigate potentially unfamiliar systems. Zhou (2023) found that students who reported higher levels of technological and academic self-efficacy were more likely to use AI-powered learning systems effectively and maintain engagement. Similarly, Zhang et al. (2023) suggested that perceived confidence in dealing with both content and technology predicts students’ willingness to explore, ask questions, and persist in problem-solving.

Self-efficacy also plays a crucial role in fostering learning interest. According to Bandura (1997), individuals are more likely to engage in and enjoy tasks when they believe they can succeed. This connection is further supported by empirical evidence indicating that students with higher self-efficacy report greater curiosity, task involvement, and emotional commitment (Du and Liu, 2017). The relationship is particularly salient in high-stress learning environments such as mathematics, where emotional states often mediate engagement behaviors. In the context of chatbot-supported mathematics learning, students’ confidence in both their academic and technological capabilities may determine whether they experience the system as helpful, interesting, or intimidating.

### 2.4 Hypotheses development

#### 2.4.1 The influence of math anxiety

Building upon the theoretical foundations established in the preceding literature review, particularly Social Cognitive Theory (Bandura, 1986) and the Unified Theory of Acceptance and Use of Technology (UTAUT) (Venkatesh et al., 2003), this study develops a series of empirically testable hypotheses. The formulation of these propositions aims to provide a robust framework for investigating the complex dynamics that influence students’ engagement and learning outcomes in technology-enhanced environments.

Drawing from the perspective of Bandura, individuals with strong self-efficacy beliefs are adept at self-regulating their learning activities, thereby effectively mitigating anxiety (Bandura et al., 1997). Empirical research consistently demonstrates that learners exhibiting higher levels of self-efficacy experience reduced academic anxiety (Unlu et al., 2017). Consequently, math anxiety is frequently conceptualized as a direct outcome or corollary of diminished mathematical self-efficacy (Hackett and Betz, 1989; Paechter et al., 2017). The presence of heightened math anxiety is thus strongly associated with lower mathematical self-efficacy, students experiencing such anxiety often harbor self-doubt and feel less confident in their mathematical abilities, which adversely affects their engagement with the subject. For instance, studies within British undergraduate populations reveal a statistically significant negative correlation between anxiety and self-efficacy (McMullan et al., 2012). Furthermore, a substantial body of research corroborates an inverse association between math anxiety, particularly as experienced during early mathematical encounters, and mathematical self-efficacy. This extensive work also reveals that math anxiety significantly impedes the development of interest in learning mathematics among the middle school students (Luo et al., 2009). This consistent evidence strongly suggests that self-efficacy tends to decline as math anxiety increases (Krapp, 1999; Nagy et al., 2006). Based on this robust theoretical and empirical background, this study posits the following hypotheses:

*H_1_* Math anxiety has a direct and negative impact on self-efficacy.

*H_2_* Math anxiety has a direct and negative impact on learning interest.

#### 2.4.2 The influence of self-efficacy

A considerable body of research consistently indicates a strong association between self-efficacy and mathematical learning interest. Specifically, mathematical self-efficacy has been identified as a partial mediator in the relationship among math interest, perseverance in learning, and academic achievement in mathematics for students of Grade eight (Du and Liu, 2017). This connection manifests as a discernible indirect effect, whereby mathematical learning interest influences academic performance via its correlation with self-efficacy and resultant grades (He and Qi, 2018). Furthermore, studies have revealed a close relationship between confidence in learning and learning interest (Gao, 1989; Ye et al., 2008), with significant associations observed for students demonstrating disinterest in learning, while moderate to low correlations apply to those who are intrinsically motivated (Chen et al., 1981; Li and Long, 2006). Self-efficacy and interest are also recognized as mutually influencing constructs (Rice et al., 2013). Within the context of the UTAUT framework, PE serves as an extrinsic motivational factor, whereas EE embodies an intrinsic one, pertaining to the process of fostering the pursuit of esteemed outcomes (Karahanna and Angst, 2006). Extensive evidence further suggests that individuals with high levels of self-efficacy often harbor favorable attitudes towards the perceived benefits and utility of computer systems (Chatzoglou et al., 2009; Gong et al., 2004; Yi and Hwang, 2003). Consequently, the following hypotheses are proposed:

*H_3_* Self-efficacy positively impacts learning interest.

*H_4_* Self-efficacy positively impacts performance expectancy.

*H_5_* Self-efficacy positively impacts effort expectancy.

#### 2.4.3 The influence of performance expectancy and effort expectancy

Within the domain of mobile learning research, PE and EE have been consistently demonstrated to play a significant role in influencing users’ adoption and usage of mobile learning platforms (Wang et al., 2009; Donaldson, 2011). Both PE and EE have emerged as pivotal factors affecting students’ engagement in online learning activities (Wang and Mao, 2016). Specifically, EE notably exerts a substantial influence within the context of mobile learning applications and Learning Management Systems (LMS) among college students (Hu et al., 2020; Raza et al., 2021). Moreover, evidence suggests a positive correlation between PE and the utilization of AI-assisted learning tools among university undergraduates (Wu et al., 2022). That is, evaluation tools based on mobile devices are capable of effectively enhancing learners’ interest in their studies (Ahmed and Parsons, 2013; Chen and Chen, 2009). Consequently, with the increasing perception of PE and EE, students’ learning experience could be improved by using AI chatbots for their math learning. Based on these considerations, the present study advances the following hypotheses:

*H_6_* Performance expectancy positively influences learning interest.

*H_7_* Effort expectancy positively influences learning interest.

#### 2.4.4 The moderating roles of students’ major and gender

**Figure 1:**
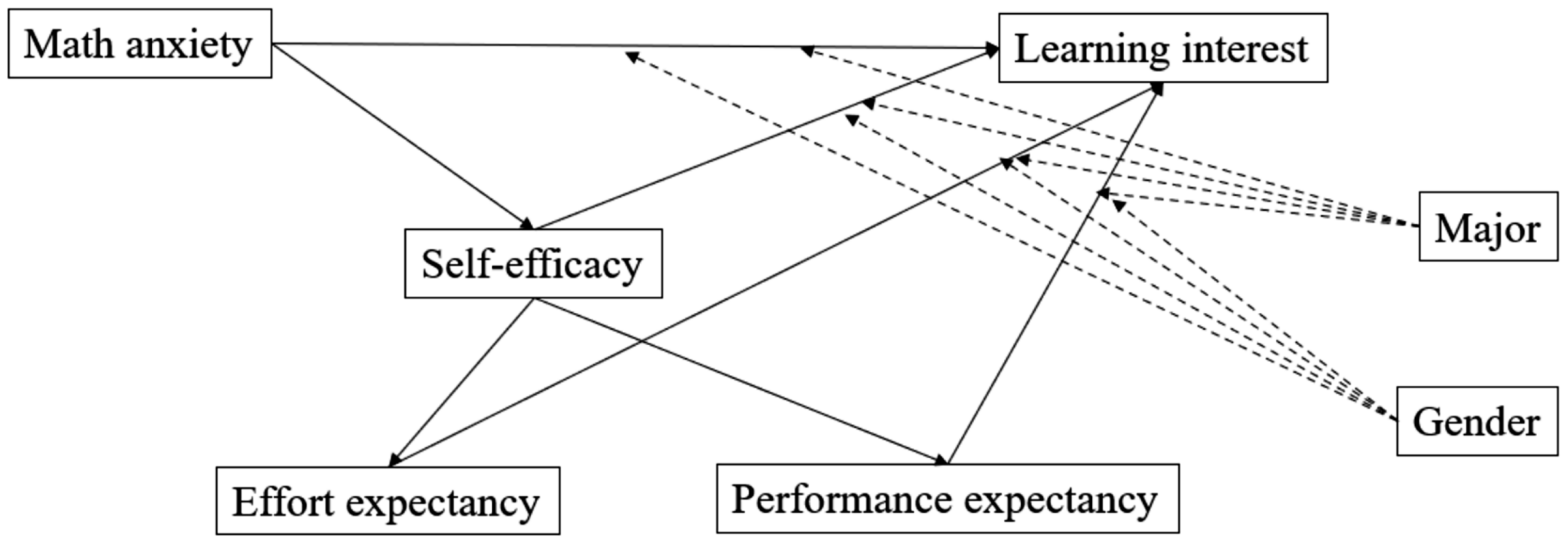
Proposed research framework.

The study by Xie et al. (2019) has indicated that there is gender discrepancy between young women and young men of Chinese high school students’ math anxiety. Moreover, the moderating effect of students’ gender has been examined in the context of math study of middle school students. More related to the present study, students’ gender has been confirmed to moderate the correlation between content quality and students’ behavioral intention towards the learning management system (Binyamin et al., 2020). Thus, the potential moderating effect of students’ gender would be examined by this study. As for the impact of students’ major, the study by Triyanto and Handayani (2018) has suggested that there is a significant difference between science and social science students in learning motivation and learning styles of higher education. Based on the this, the potential moderating of students’ major would be examined. Figure 1 presents the proposed research framework.

## 3 Method

### 3.1 Measures and questionnaire

The initial section of the study outlines the key demographic characteristics of the participants, including gender, field of study, and academic year. Following this, the study proceeds to operationalize and measure the core constructs of the proposed research model.

The measurement instruments used in this study were adapted from well-established and validated scales. The Mathematics Anxiety Rating Scale (MARS) developed by Richardson and Suinn (1972) served as the foundational instrument for assessing mathematics anxiety. Based on this, a simplified version was adopted, which differentiates between anxiety related to math learning and math testing (Plake and Parker, 1982). The scale developed by Li and Tian (2014) for high school students further subdivides math anxiety into three dimensions: exam anxiety, anxiety during classroom learning, and anxiety during extracurricular problem-solving. While various international versions of math anxiety scales differ in their focus depending on target populations, common dimensions typically include exam-related, learning-process, and numerical anxiety. Drawing upon these, the current study measured math anxiety across three subdimensions: math test anxiety, math task anxiety, and math course anxiety. Mathematical self-efficacy was measured using a revised version of the Academic Self-Efficacy Scale developed by Pintrich and Groot (1990). In this study, self-efficacy was divided into two dimensions: self-efficacy in learning ability and self-efficacy in learning behavior.

In addition, PE and EE were adapted from the UTAUT model to assess students’ experiences of using AI chatbots as learning tools for mathematics. All questionnaire items were rated using a 7-point Likert scale, with values ranging from 1 (“strongly disagree”) to 7 (“strongly agree”). Moreover, because the research was conducted in Mainland of China, thus, the questionnaire was developed in Chinese at the first beginning. Afterwards, the ethics committee of the university of the author that works at has approved the questionnaire and corresponding questions contained. The developed questionnaire has been reviewed by three experts in the field and three undergraduate students. The minor revisions have been made to ensure the accuracy and appropriateness of the questionnaire. The final agreement has been made among the panel.

### 3.2 Sample and data collection

The questionnaire was designed and distributed using the online platform Questionnaire Star, known for its accessibility, low cost, and ease of participation (Jin et al., 2021; Su et al., 2021). The primary means of distribution was through social media platforms, such as WeChat. It is important to note that participants received no financial compensation for their involvement in this study.

The questionnaire was disseminated across various provinces and cities in China, with particular emphasis on the cities of Hangzhou, Jiaxing, and Shaoxing in Zhejiang Province, targeting undergraduate university students. At the first beginning of the questionnaire, the informed consent form has been announced and if the potential respondents allowed to answer the questionnaire, it could refer that the consent form has been approved by respondents. The notification of the academia-use-only and confidential of respondents’ information have been promised to the respondents.

A total of 401 responses were collected, from which 247 valid responses were retained. Inclusion criteria required a minimum response time of 90 s, and responses exhibiting uniform or extreme patterns were excluded. This yielded a final effective response rate of approximately 61.6%. Among the valid respondents, 121 were female (49%) and 126 were male (51%).

A majority of participants were enrolled in science and engineering disciplines (42.9%), followed by natural sciences (23.9%), humanities and social sciences (21.1%), and others (12.1%). In terms of academic year, sophomores and juniors made up 71.2% of the sample, while freshmen accounted for 15.0%, seniors for 12.6%, and others for 1.2%. A detailed breakdown of the sample’s demographic characteristics is presented in Table 1.

**Table 1:** Respondents’ characteristics.

Measure	Item	Frequency	Percentage
Gender	Female	121	49.0
	Male	126	51.0
School	Humanities and Social Sciences	52	21.1
	Natural Sciences	59	23.9
	Science and Engineer	106	42.9
	Others	30	12.1
Grade	First grade	37	15.0
	Second grade	113	45.7
	Third grade	63	25.5
	Fourth grade	31	12.6
	Others	3	1.2

### 3.3 Data analysis

For data analysis, the statistical software packages SPSS and SmartPLS were used, with the study employing Partial Least Squares Structural Equation Modeling (PLS-SEM) to analyze the relationships among latent variables. Specifically, SmartPLS was used to evaluate both the measurement model and the structural model (Silva et al., 2014). The measurement model was tested for validity and reliability to assess the causal relationships among latent constructs. PLS-SEM is well-suited for exploratory research, non-normal data distributions, and small sample sizes. Unlike covariance-based SEM (CB-SEM), PLS-SEM places fewer restrictions on data normality and is capable of estimating complex models even with modest sample sizes. Typically, a sample size should be at least 10 times the number of the most highly loaded indicators in the model (Wolf et al., 2013). Moreover, some empirical studies suggest that a minimum sample size of 200 is a more conservative and reliable approach for a buffer against potential model estimation issues (Kock, 2018). In this study, the construct math anxiety included 13 indicators, and the sample size of 247 was therefore sufficient for model estimation under these two rules. This method also enables robust estimation of structural relationships and is particularly effective for theory development in emerging fields (Haenlein and Kaplan, 2004; Hair et al., 2019; Hair et al., 2021; Shiau et al., 2019).

Specifically, to evaluate the measurement model, item loadings, reliability, convergent validity, and discriminant validity were examined in accordance with the guidelines proposed by Hair et al. (2021). Reliability pertains to the internal consistency and stability of measurement scales. Generally, Cronbach’s alpha values above 0.70 indicate acceptable reliability, with values closer to 1.0 reflecting higher internal consistency (Hair et al., 2021). As for the convergent validity, AVE values above 0.50 are considered ideal (Fornell and Larcker, 1981). Moreover, the value of composite reliability (CR) higher than 0.7 was also recommended (Hair et al., 2021). Furthermore, discriminant validity was assessed using the Heterotrait-Monotrait ratio (HTMT) and cross-loadings. Following Henseler et al. (2015), HTMT values below 0.90 indicate acceptable discriminant validity. As for the cross loadings, it is supposed that the loadings of every item should be higher than the loadings of its equivalent variable (Gefen et al., 2000). At last, the Standardized Root Mean Square Residual (SRMR) would be applied to testify the model fit and the cut-off value was determined to be lower than 0.08 (Henseler et al., 2015).

## 4 Data analysis and results

### 4.1 Results of measurement model

Individual item reliability was first examined by analyzing factor loadings. The composite reliability (CR), average variance extracted (AVE), and Cronbach’s alpha values for each construct were computed using the PLS algorithm in SmartPLS, as presented in Table 2. In this study, all constructs achieved satisfactory reliability, with the lowest Cronbach’s alpha being 0.801. In addition, all composite reliability values exceeded 0.883, and AVE values were above the threshold of 0.50, with the lowest being 0.588. These results indicate adequate construct reliability and convergent validity. Item factor loadings were also inspected, and all values exceeded the recommended minimum of 0.40 for exploratory studies (Hair et al., 2022), with the lowest loading being 0.642. For the discriminant validity, all HTMT values fell within acceptable ranges of lower than 0.9, confirming the distinctiveness of the constructs which was scrutinized in Table 3. Moreover, for the criteria of cross-loadings, it was examined that each item external loading on its associated construct is greater than its cross-loading on other constructs, indicating the discriminant validity has been met by this measure.

**Table 2:** Item loadings, CRs, and AVEs of measurement model.

Construct	Item	Loading	CA	CR	AVE
LI	li1	0.867	0.899	0.930	0.768
	li2	0.861			
	li3	0.878			
	li4	0.900			
MA	ma1	0.771	0.942	0.949	0.588
	ma2	0.773			
	ma3	0.824			
	ma4	0.762			
	ma5	0.737			
	ma6	0.748			
	ma7	0.770			
	ma8	0.828			
	ma9	0.752			
	ma10	0.806			
	ma11	0.642			
	ma12	0.856			
	ma13	0.671			
EE	ee1	0.872	0.801	0.883	0.716
	ee2	0.883			
	ee3	0.780			
PE	pe1	0.770	0.883	0.915	0.683
	pe2	0.861			
	pe3	0.863			
	pe4	0.816			
	pe5	0.819			
SE	se1	0.715	0.893	0.918	0.653
	se2	0.802			
	se3	0.800			
	se4	0.875			
	se5	0.809			
	se6	0.839			

LI: learning interest; MA: mathematics anxiety; EE: effort expectancy; PE: performance expectancy; SE: self-efficacy; CA: Cronbach’s alpha; CR: composite reliability; AVE: average extracted variance.

**Table 3:** HTMT values.

Construct	LI	MA	EE	PE	SE
LI	0.877				
MA	0.342	0.767			
EE	0.775	0.361	0.846		
PE	0.769	0.366	0.832	0.826	
SE	0.537	0.058	0.530	0.512	0.808

LI: learning interest; MA: mathematics anxiety; EE: effort expectancy; PE: performance expectancy; SE: self-efficacy.

### 4.2 Results of structural model

The structural model was evaluated by examining collinearity (VIF values), path coefficients, effect sizes (*f^2^*), coefficient of determination (*R^2^*), and predictive relevance (*Q^2^*). Bootstrapping with 10,000 subsamples was used to assess the statistical significance of the path coefficients. Table 4 summarizes the results of hypothesis testing.

**Table 4:** Path coefficients and the results of the significance tests.

H	Path	*β*	*t*-value	*p* value	Decision
H_1_	Math anxiety → self-efficacy	0.058	0.617	0.537	Rejected
H_2_	Math anxiety → learning interest	0.067	1.712	0.087	Rejected
H_3_	Self-efficacy → learning interest	0.171	2.873	0.004	Supported
H_4_	Self-efficacy → performance expectancy	0.512	7.574	<0.001	Supported
H_5_	Self-efficacy → effort expectancy	0.530	8.377	<0.001	Supported
H_6_	Performance expectancy → learning interest	0.370	4.579	<0.001	Supported
H_7_	Effort expectancy → learning interest	0.346	4.452	<0.001	Supported

Out of the seven proposed hypotheses, five (H3, H4, H5, H6, and H7) were supported at a significance level of *p* < 0.05, while two (H1 and H2) were not supported. Notably, self-efficacy showed a significant positive impact on learning interest, PE and EE, while both PE and EE had significant positive effects on learning interest.

The moderating effects of gender and academic major were examined and are reported in Table 5. The results indicate that academic major significantly moderated the relationship between math anxiety and learning interest, while no other moderating effects reached statistical significance.

**Table 5:** Result of the moderating effects.

Path	*β*	*t*-value	*p* value	Results
Gender × math anxiety → learning interest	−0.000	0.008	0.994	No
Gender × self-efficacy → learning interest	−0.109	1.678	0.093	No
Gender × performance expectancy → learning interest	0.059	0.704	0.481	No
Gender × effort expectancy → learning interest	0.042	0.538	0.591	No
Major × math anxiety → leaning interest	−0.108	2.534	0.011	Yes
Major × self-efficacy → learning interest	0.002	0.033	0.974	No
Major × performance expectancy → learning interest	−0.043	0.557	0.557	No
Major × effort expectancy → learning interest	0.029	0.374	0.708	No

Collinearity was evaluated using VIF values, and all path-related VIF scores were below the threshold of 5.0 (see Table 6), indicating no multicollinearity concerns.

**Table 6:** Result of the variables’ collinearity indicators and the intensity of the effect.

Path	*f^2^*	VIF
Math anxiety → Self-efficacy	0.003	1.000
Math anxiety → Learning interest	0.011	1.351
Self-efficacy → Learning interest	0.059	1.614
Self-efficacy → Performance expectancy	0.355	1.000
Self-efficacy → Effort expectancy	0.390	1.000
Performance expectancy → Learning interest	0.126	3.520
Effort expectancy → Learning interest	0.105	3.676

Effect sizes (*f^2^*) were also computed. Based on the benchmarks provided by Hair et al. (2021), values above 0.02, 0.15, and 0.35 indicate small, medium, and large effects, respectively. The largest effect size was observed in the relationship between self-efficacy and EE (*f^2^* = 0.390), followed by self-efficacy → PE (*f^2^* = 0.355), EE → learning interest (*f^2^* = 0.126), and EE → learning interest (*f^2^* = 0.105).

Model fit and predictive power were assessed using *R^2^*, *Q^2^* and SRMR. As shown in Table 7, the *R^2^* value for learning interest was 0.692, indicating substantial explanatory power. The *R^2^* values for EE (0.281) and PE (0.262) suggest moderate explanatory strength, while self-efficacy showed low *R^2^* (0.003). In terms of *Q^2^*, learning interest achieved a value of 0.515, signifying strong predictive relevance, whereas EE (0.198) and PE (0.175) showed moderate levels. Self-efficacy again exhibited weak predictive relevance (*Q^2^* = 0.002). The SRMR value was 0.062, which is below the acceptable cutoff of 0.08, thereby confirming that the model fits the data well. In summary, the empirical findings support five of the seven hypothesized relationships. EE and PE emerged as the strongest predictors of learning interest, validating the central role of technology-related beliefs in shaping students’ motivation to engage with AI chatbots in mathematics learning.

**Table 7:** The predictive power of structural model.

Construct	*Q^2^*	*R^2^*
LI	0.515	0.692
EE	0.198	0.281
PE	0.175	0.262
SE	0.002	0.003
SRMR = 0.062		

LI: learning interest; MA: mathematics anxiety; EE: effort expectancy; PE: performance expectancy; SE: self-efficacy.

## 5 Discussion

With the expansion and popularity of AI chatbots in the higher education, this study explores the formation mechanism of undergraduate students’ learning interest to math with AI-empowered learning. By incorporating constructs from both the UTAUT model and Social Cognitive Theory, the study was conducted with empirical data of 247 respondents from college. Consequently, it was revealed that EE (*β* = 0.346. *t* = 4.452, *p* < 0.001) and PE (*β* = 0.370. *t* = 4.579, *p* < 0.001) were the strongest predictors of the formation of students’ learning interest. Moreover, self-efficacy also demonstrated a significant impact on students’ learning interest (*β* = 0.171. *t* = 2.873, *p* < 0.01) as well as positively impact on students’ perception of PE (*β* = 0.512. *t* = 7.574, *p* < 0.001) and EE (*β* = 0.530. *t* = 8.377, *p* < 0.001). These findings affirm the central role of EE and PE which are the two critical dimensions of the UTAUT framework, in shaping learners’ motivational engagement with AI-enhanced educational tools. Overall, the results concluded in this study, could to some extent, contribute to the growing body of literature on AI integration in education by highlighting how chatbot technologies can serve as a bridge between learners’ psychological readiness and their affective engagement with mathematics. While prior studies have focused on learners’ acceptance of e-learning systems or mobile learning platforms, this study expands the conversation to AI chatbot-supported learning, confirming that established technology acceptance variables remain relevant in this emerging context. Specifically, the findings suggest that when students perceive chatbot interactions as easy and beneficial, they are more likely to develop interest in the learning process even in a subject as anxiety provoking as mathematics.

The significant effect of self-efficacy further underscores the motivational foundation of technology-assisted learning. Students with higher self-efficacy are more confident in navigating AI-supported tools, which in turn reinforces their positive evaluations of these tools’ utility and usability. This supports Bandura’s theoretical proposition that efficacy beliefs shape not only learners’ task engagement but also their openness to adopting novel strategies and technologies.

However, the study also yielded several unexpected findings that warrant further reflection. Math anxiety, a variable extensively studied in educational psychology, was found to have no significant influence on either self-efficacy or learning interest in the present model. This result contrasts with much of the existing literature, which typically reports negative associations between anxiety and motivation-related constructs. One possible interpretation is that the presence of AI chatbots served as a psychological buffer, distancing students from the traditional sources of anxiety (e.g., teacher judgment, peer comparison) and thereby reducing its direct influence on their confidence and interest. This has been discussed by previous studies. For instance, Hsu et al. (2023) have investigated the decision-making chatbot with students’ relative knowledge could significantly improve their learning achievement and enjoyment, meanwhile, their anxiety could be reduced among high school students. Moreover, the empirical investigation among students in the Islamic Religious Education (IRE) program has suggested that that students’ learning interest is positively associated with their usage of artificial intelligence (AI) (Fauzi et al., 2025). It is also possible that students reinterpreted the learning context as being less threatening when mediated by technology, thus diluting the emotional intensity normally associated with math-related tasks. In terms of the moderating effects of students’ gender and major, only one path from students’ math anxiety and their learning interest has been examined to be moderated by students’ major (*β* = −0.108. *t* = 2.534, *p* < 0.05), suggesting that there is discrepancy between students’ major for the formation of their learning interest from the impact of their math anxiety.

## 6 Conclusion

This study constructed and empirically validated a structural model to examine the factors influencing undergraduate students’ learning interest in AI chatbot-assisted mathematics education. Drawing on the Unified Theory of Acceptance and Use of Technology (UTAUT) and Social Cognitive Theory, the findings revealed that EE, PE, and self-efficacy were all significant predictors of learning interest, with EE demonstrating the most substantial influence. These results underscore the critical role of perceived ease of use and user confidence in fostering engagement with intelligent learning systems.

Notably, self-efficacy not only exerted a direct effect on learning interest but also served as an antecedent to students’ perceptions of chatbot utility and usability, highlighting its mediating role in technology-enhanced learning. The findings also revealed that math anxiety had no significant direct impact on either self-efficacy or learning interest, a departure from traditional expectations. This result may reflect the buffering effect of AI-mediated learning environments, which potentially mitigate the affective constraints typically associated with mathematics.

Furthermore, the moderating effect of academic major suggests that disciplinary context shapes the relationship between affective and motivational variables. In fields where mathematics is central to academic identity, anxiety may be internalized differently than in non-STEM disciplines, thereby influencing the salience of motivational constructs. These results collectively reinforce the view that learners’ emotional, cognitive, and technological experiences are deeply intertwined, and that these relationships are context-dependent.

### 6.1 Theoretical contribution

This research offers three principal theoretical contributions. First, it extends the scope of UTAUT-based research by shifting the outcome variable from behavioral intention to learning interest, thus integrating motivational dimensions more explicitly into technology acceptance research. This shift bridges the traditionally separate domains of educational psychology and technology acceptance literature which could provide a research paradigm for the future research to investigate the relevant subject learning with the assistance of AI. Second, the study positions AI chatbots within the under-researched domain of mathematics education, offering a novel application scenario for intelligent tutoring systems. While prior research has largely focused on chatbots in language learning or generic instructional roles, this study demonstrates their relevance and pedagogical potential in anxiety-prone, cognitively demanding domains (la De Vall and Araya, 2023; Son et al., 2025). Third, by incorporating self-efficacy as both a direct and indirect driver of learning interest, the study highlights the importance of learner agency and self-perception in mediating the adoption and emotional reception of AI-driven technologies. This finding enriches the theoretical dialogue between cognitive-affective models and technology-enhanced learning frameworks.

### 6.2 Practical implications

The study yields several actionable insights for educators, instructional designers, and technology developers. Foremost, the ease of interaction with AI chatbots emerges as a decisive factor in stimulating students’ academic interest. Thus, user-centered design and intuitive system interfaces should be prioritized these characteristics in the development of educational chatbot. That is, the ease of AI chatbot could to some extent, boost students’ learning interest. Additionally, interventions aimed at enhancing students’ academic self-efficacy may have downstream effects on their motivation and openness toward AI-assisted learning environments. Institutions may consider integrating AI-based tools with pedagogical scaffolding to foster both technological acceptance and subject-matter engagement. Finally, instructional practices should be sensitive to the disciplinary context and emotional profile of learners. In mathematics education, where anxiety is prevalent, the strategic use of supportive AI systems may reduce psychological barriers and cultivate positive learning dispositions.

### 6.3 Limitations and directions for future research

Several limitations warrant acknowledgment. First, the cross-sectional nature of the study limits causal inference. Future longitudinal or experimental research designs could better capture the dynamic interplay between psychological constructs and learning interest over time. Second, the sample was geographically restricted to universities in Zhejiang Province, which may limit generalizability. Broader cross-regional and cross-cultural sampling would enhance the external validity of the findings.

Third, the study focused exclusively on mathematics learning. Future research could examine whether the identified patterns hold in other subject areas, particularly those with different cognitive and emotional demands, such as language learning or the arts. Finally, future investigations could explore additional moderating and mediating variables, such as digital literacy, prior experience with AI systems, or learners’ epistemological beliefs, to better understand the complex mechanisms underlying technology-mediated motivation.

## Data Availability

The raw data supporting the conclusions of this article will be made available by the authors, without undue reservation.
